# Memorial Address upon the Life and Work of Adolph Lippe

**Published:** 1888-11

**Authors:** P. P. Wells

**Affiliations:** Brooklyn


### MEMORIAL ADDRESS UPON THE LIFE AND WORK OF ADOLPH LIPPE.

P. P. Wells, M. D., Brooklyn.

(We give here only a condensed report of the Memorial Address delivered October 29th, by Dr. P. P. Wells at the Women's Homœopathic Hospital of Pennsylvania. We are sorry space will not allow us to give this excellent address in full. We believe it is the intention of the ladies of the Memorial Association to publish the address in full for distribution, so all may read it.)

It is well, when those who have been largely gifted with powers for doing good in their generation, have passed from us to the untried world, that they should be retained in the memory of those who survive, to whom must be committed the work they have laid down.

It is proper that such persons should be held in memory on the lines on which they thought and acted. It is well that these lines of thought and action, and the principles of philosophy which guided both, should be clearly recognized and ever held in plain view, by all who are ambitious of the greatest possible successes in practical life. Such may rest perfectly assured that the results which crowned the life and labors of Adolph Lippe, rendering them glorious, and worthy of our memory, can only be attained by work on the lines he followed, and in perfect obedience to the philosophy to which his heart and life were given, with such perfect loyalty and obedience. It should never be forgotten by faithful hearts, that there is no short and easy method to the results which attend loyalty to law, and obedience to its practical methods. To attempt to gain these by shorter and easier methods is only to dishonor law and secure disgrace which attaches to failures.

When those so gifted and so active lay down their work, it is good that the memory of their work be cherished and kept alive in the hearts of those who remain to take up and carry forward the work which death has committed to their hands. To secure this object it has been common to erect some material object suggestive of the man, his virtues, or his work, so that memory may be quickened and emulation of the virtues and good deeds of those so memorialized may be excited.

In erecting such a monument to the great and good in order to secure this objective, it is indispensable that there be in the memorial, in its nature or material, a suggestion of the man or his work which shall excite and maintain the sympathy of the beholders with the man and work memorialized. It is at once recognized that the same monument could not suitably represent the eminent warrior, statesman, or divine. The monument to each must be suited to the character and work of the man it would call up in our memory; it must be such as will excite in us sympathy with the excellence of that character and work. In this view, what shall be found in monumental form, which will most vividly recall to our minds the friend and teacher who has left us, and left us without a successor? What shall it be which will most forcibly revive the memory of his great practical life work, the results of which the most ambitious may and will be satisfied with, if he can equal in his practical labors? What shall be the monument raised to the memory of Adolph Lippe, and what are the reasons which call for its erection? We will, for a moment, look at these two questions.

We will examine the second question first. The reason why we should retain in our memory the name and the work of Adolph Lippe? First, because he was a man with convictions of truth in the matters of his life work, and with a courage which enabled him to carry these loyally into his daily duties. Whatever of opposition, ridicule, or reproach there may have been brought upon him by reason of these, what he accepted as truth he manfully maintained, defended, and obeyed. Whatever he met of opposition or reproach, because of these, he was ever able to withstand and to treat as they deserved, either with unanswerable argument, or a never weakening contempt. The petty wit, sarcasm, and ridicule, vain ignorance and empty conceit cast on him and the truth he had accepted and practiced, he could pass by as less than vanity, knowing well the character of its origin, and if the persistency and iteration of these sometimes excited his wrath they never caused him to swerve from the truth he loved and had made the foundation of his life work. How irritating these sometimes were, those who are old enough to remember when the practitioners of Homœopathy were few, may well remember; those who are younger, and only remember when these practitioners have become many, can have no realizing sense of this, and if we add to these the falsehoods and misrepresentations, which usually accompanied the opposition our earlier *confrères* met, we may not be greatly surprised if we remember our friend sometimes (as the great master whom he venerated and followed did before him) lost his patience and his temper when he saw truth so treated by those who knew no better.

Second, he was loyal to the law he accepted; as God given, he recognized the extent of the relationship of this law. Its origin in infinite wisdom and benevolence was to him a sufficient guarantee of its universality of relationship to all curable sicknesses; that intelligent and loyal administration of this law assured the greatest possible release to the pains and dangers incident to sickness of whatever form. This view of the extent of the law, and conviction of its supremacy over suffering and death, bound him to it in a spirit of perfect confidence and consecration. Who will say he was wrong if he regarded those who would reduce the universal breadth of this law to a "narrow groove," or to the limits of a "mere dogma," as no better than enemies of God and man, and dealt with them as he thought such renegades or reprobates deserved? Is it remarkable if this dealing at times had in it little of patience or forbearance?

He was equally clear in his recognition of the inseparable corollaries of this law. He saw their importance in the clearest light, and that compliance with their demands was equally necessary to success in treating as obedience to the law of similars. The first of these is the starting-point for every endeavor for a cure of any sickness: "the totality of the symptoms;" this is declared to be "*the sole indication in the choice of the remedy.*" This declaration our friend accepted as truth, not to be questioned, and placed it as the chief corner-stone in the foundation principles of his life work. He gave to no associate science of therapeutics part with this corollary in its execution of its specific office. If called to account by those less intelligent than himself in the science and art of specific healing, because the acceptance of this corollary gave sole authority in this choice to this totality, thus excluding from this duty the highly prized science of pathology, he would at once reply, this totality is the only science of pathology known to specific medicine in the choice of its curatives; and he might charge back on his materialistic questioner, that all the knowledge he possesses of his unseen imagined internal something which he calls pathology is in this totality, and beyond this all, as to internal condition in cases of sickness, is as dark as Erebus even to him who assumes to know most. He can know only what symptoms disclose to him. One remedy at a time was as necessary to legitimate results of specific prescribing as was the selection of the most similar remedy, and this because if two were given no man could foretell the effect of each on the other, and of the combined action of the two in these circumstances, as no knowledge of this has been obtained by any experiments or observations. The assumption that this will be made up of the action of one plus that of the other, as these appear in the record of their provings, is fully gratuitous, and has no confirmation in any intelligent experience.

One thing, however, may be safely predicated of this violation of the second corollary of our law—a confusion of phenomena in the sickness which no intelligence may be able to unravel or remedy. It will be seen that, for these reasons, obedience to this corollary is fatal to that senseless heresy (practiced by those who do not know the law or the specific curative when they see them) called "alternation." This has its origin wholly in the uncertainty in the mind of the prescriber. He does not know, and hence is not quite sure, whether A or B is the remedy, or, indeed, that either of them is, and so, to make certain of the greatest possible good, he will give both, though he knows nothing of the effects of those drugs when so given. He will, most likely, fail of the greatest possible good, and he is all too likely to produce the greatest possible confusion and embarrassment.

The third corollary of the law was equally recognized and accepted as authoritative by our friend. The least possible of the remedy which the cure requires was the thought, ever present with him with the power and authority of truth, whenever he administered drugs for the relief of the sick. The necessity of this rule he saw clearly in the nature of things; he saw that all drugs are sick-making agents. That the most similar remedy, the specific curative of the case, makes sick in the manner and direction of the acting morbid cause. Hence, all of the drug agent given, more than is required to neutralize the action of the morbid cause, can only add to the intensity of the action of that morbid cause, and so to increase both the suffering and the danger of the patient. Much and great evil has come from disregard of this corollary, and the more accurate has been the selection of the remedy the greater has been the harm from over-dosing.

This corollary of our law, "*The minimum dose of the dynamized drug*," it will be seen, is made up of two elements—the amount of the agent given and the state in which it is to be administered. The two are not founded on the same basis; the amount is a logical outcome of the nature of the portions which constitute each clinical problem the prescriber of specific medicine is called on to solve. It is different with the second element, that of the state in which the similar remedy shall be given to the patient; this is in no sense or part a result of logic or of reasoning, but is wholly a child of experience. It was accepted and made an authoritative part in the life work of our friend, because the experience of the great masters of our healing art, himself included in the number, had demonstrated that in the dynamized state the most similar remedy is curative in a far greater degree than when administered in the crude form. The truth of this had been so many thousands of times demonstrated to his observation that no experience of his practical life was received with more confidence than this, of intensified curative force of the drug resulting from the process of dynamization. And because of this ascertained increase of power to cure, in this state, the requirement in this corollary, that it be so given, has become a demand of law in our administration of specific therapeutics.

The demand of this law and its corollaries, on those who would practice the system of specific medicine taught by Hahnemann, for intelligent acceptance and obedience was responded to by our friend in perfect good faith. It was this response which placed him in the highest rank of healers, where he stood with few equals and still fewer superiors.

Dr. Lippe's intelligence went far beyond a mere acceptance of the truth of our therapeutic law and its corollaries. It apprehended clearly the true nature of the factors involved in every problem for therapeutic solution—the true nature of sickness, and that of the agent by which sickness is cured. He saw clearly the error of those who regarded sickness as entities, as things distinct from the sufferer they are to care for, and that those who claimed exact knowledge of these things, as to locality, form, and nature, were only deceivers or deceived. That the dignifying of this claim by attaching to it so respectable a word as pathology in no way gave it aught of value; that this kind of pathology added nothing to any man's power as a healer.

Instead of accepting sickness as a material thing, he saw them only as disturbances of the action of the force which moves and controls the functions of the organs of the living body, so that the harmony of these functions which conserves the organism in whole and in all its parts which constitutes health, is lost, and is replaced by the discord; sickness, then, is not a thing but a disturbed force. Not a material entity, but an immaterial force, perverted as to its moving and governing of bodily functions. So he saw sicknesses to be, and he saw them just as they really and always and only are. Then it follows that a true science of pathology must of necessity be, and only be, made up of a knowledge of those disturbances of life-force and functions which we are accustomed to speak of as "*the totality of the symptoms*;" this is the only true pathology of any case. So, when it was alleged against Hahnemann, by his materialistic antagonist, that he had no pathology in his system of specific medicine, he alone of all the world had and taught a pathology which had its foundation in truth and the nature of things. The true philosophy, then, of sickness is a philosophy of dynamics—of form and its perverted action on functions. This philosophy was accepted by Dr. Lippe, and, together with his loyalty to law and its corollaries, helped to raise him to his height of excellence as a healer.

He saw, also, the true nature of the ætiology of diseases, and that this was in perfect harmony with the accepted philosophy of the law, its corollaries, and the nature of the result its causes produce; that these causes, like the sickness man is called to cure, were not of mechanical or chemical origin, but of dynamic nature. The truth of this is demonstrated in all miasmatic causes and contagions. These are wholly beyond the grasp of human intelligence, except as their existence is revealed in the phenomena resulting from their assaults on the life-force of man. They are wholly beyond the reach of the microscope or of the most enlightened chemist, and are as far removed from the much-talked-of living "germs" of the material ætiologist as is his promised protection by the destruction of the "germs" from truth. The causes of malarial fevers, malignant cholera, variola, etc., are as inscrutable, except as studied in the result of their action on the life-force, as are the thoughts of the angels in Heaven. Hence the folly of much of the talk of disinfection and the proceedings with germicides for the accomplishment of this end. And hence has come so much disappointment after liberal use of so-called "disinfectants" which do not disinfect. Our friend was equally intelligent as to the nature of the agents which were to be used in human sicknesses when administered in compliance with the demands of law. He accepted the truth, many thousand times demonstrated in his own experience, that this power, which comes, is not from the matter of the agent at all, but a dynamic force associated with that matter, which, by the manipulation taught by Hahnemann, could be liberated from its original association, and so made available to specific medicine for the cure of human sicknesses. That substances apparently inert were made to yield a dynamic of great power to heal, while the most destructive poisons were caused and converted into beneficent healing agents by this process, called dynamization.

It is not at all surprising that the materialistic antagonist of Hahnemann's medical philosophy was only distracted when told of cures of serious sicknesses wrought by these almost demonstrated nonentities. Do mathematical demonstrations lie? Can something come from a demonstrated nothing? Very likely not. But the falsehood of this otherwise correct reasoning comes from its starting-point. It takes for granted what can never be proven: that the power which cures is identical with the matter of the drug, while every cure wrought by these dynamizations is a demonstrated truth that it is not. It further exposes the folly of attempts at reasoning from matter and mathematics on a subject with which neither are in any way related.

We have very briefly given a view of the principles which give individuality to Adolph Lippe as a healer, and to his life work as one of eminent successes. These principles were so clearly recognized, and accepted so unreservedly, that they virtually became a part of his own life. They were ever present in his thoughts, and dominated every decision in his clinical work. And before each problem of curing he was called to solve, his first inquiry was: What does the law demand for the relief of this *one* case? and he earnestly and honestly set himself to answer this demand, for in all his practical life he was a servant of law. It was never first in his thoughts before a knotty and complicated case: *how shall this be named?* but, rather, what does the law require for its cure, and how shall this be found? What! says old physic, go for a remedy before you have made your diagnosis? Why, if the remedy proves a success, you never know what you have cured! I think I hear my friend reply to this very foolish criticism: I cured my patient, and this was the objective of my endeavor, and neither nature nor law—its law of healing—required a Greek name to make his cure complete.

It is, then, not Adolph Lippe, the man whose memory you would perpetuate, but Adolph Lippe the great healer of men. As a man he was like other good citizens, discharging the common duties of life faithfully; as a healer of sick men he stands out conspicuous in the ranks of those who stand highest. With natural endowments qualifying him for the work of his choice of a very high order, he brought to this work a consecration of heart and life under the control, and in the light of law. Loyalty to law was the one governing principle in his work which made it phenomenal by reason of its many and great practical successes. This loyalty had its origin in clear perception of the origin of the law of healing, of its logical connection with other laws having the same origin, and that, like other natural laws, this also was of universal application and authority. The voice of man—no matter what his reputation for intellectual power and knowledge of the natural sciences—what is this when opposing the speech of the Supreme Omniscient. It had no influence to reduce our friend from his confidence in the divine speech, or for a moment to cause him to waver in his integrity to divine commands. The law was to him not a "*rule*," but a command.

Is it a memory of this healer, so constituted in his nature, so consecrated in his spirit to truth and law, so unyielding to whatever of influence of learned men of great reputation in the world of the sciences, so quick to detect, and so powerful to rebuke error, that you would keep alive in the hearts and minds of those who survived him? Then by what means can this best be done? Fitness in the means employed for this purpose is necessary for the attainment of the objective of the memorial. The memory of a Washington or a Lincoln may be perpetuated by a shaft or statue. This has been done by both, and the sense of propriety in man has been abundantly satisfied by each. A statue may fitly suggest greatness in the orator or the jurist, but neither of these has any voice to remind us of the man who was eminent among men as a healer of their sicknesses and pains. They are in their very nature dumb as to all which gave the man his great power for good. They are wholly blind as to any recognition of principles of truth in law, or in the relationship which law has established between sicknesses and the agents by which their healing shall be effected. No material monument can be erected which can be at all expressive of these. Such monuments may express ideas of material facts or existences, but for expression of the dynamic nature of sicknesses and their curatives these have no power.

They are equally imbecile before the problem of representing the skill which has rightly selected, rightly managed the administration of the means which law has demonstrated for the successful healings which have made the life of the man you would memorialize so conspicuous. This man was truly great in the work to which he gave his thoughts and his life, but no material form can fully represent the principles which inspired his life work, or the loyal and unswerving obedience which gave the success which made him great among healers.

Then, if we are to memorialize this man and his life work, we must look for the means beyond the common marble and bronze, which may fitly enough represent to succeeding generations the character and work of the warrior, the statesman, or jurist or orator, but can only be found dumb and incongruous when the question is of perpetuating the memory of the man and his life work, when this has been wholly given to the relieving of suffering and sickness of man. A single act, as that found in the story of the good Samaritan, may be successfully treated by the artist with brush or chisel, but when it is a life devoted to truth in philosophical principles and to a daily demonstration of their truth and beneficence, we are compelled to look for means by which fitly to represent and bring to our mind such a life and such a work beyond the material things of the world. We can only find fitting memorials of such a life and work in living examples which shall repeat the work and experiences of the man to be memorialized.

The method of presenting these examples which seems fullest of promise of success in keeping alive the memory of our friend and his work, and to contain in it most of good for mankind, is to teach others the principles, methods, and means which characterized his work, and gave to him the many and great successes which make him conspicuous in the ranks of great healers. If he could have been consulted as to the kind of monument by which he would prefer to be retained in the memory of men, he would, no doubt, have chosen an organization for teaching the truths and principles which he so firmly believed, so dearly loved, and to which he wasso truly and loyally obedient. He would have said, "These faithfully and truly taught, and my spirit will be fully satisfied. Let these be taught to others, that they may be able to bless men by reason of their knowledge of the truth as revealed to Hahnemann, and by him given to us, by which truth I have gained all of the successes which have attended my labors."

Then, to satisfy the spirit of our friend, let it be remembered, these truths must be taught as he himself would have taught them. Freedom from all mixture, from whatever source, of tradition or hypothesis intruded into the companionship of these truths, as improvements of the divine law he so unswervedly accepted, must be most carefully maintained. These, wherever met, were to him a horror. It was no relief to him, rather an abomination, when these intruders were presented as evidence of progressive thought, or as added improvements of law. They were to him always and only embarrassments to all right progress, and were ever recognized and treated as the work of the arch enemy.

This is the kind of law to be taught in any attempt to memorialize the work, life, and successes of Adolph Lippe.

And, more than this, the logical corollaries of this law are to be taught no less faithfully, and as of authority equal to that of the law itself. There are to be no perhapses or ifs in the acceptance of these as supreme in their control of all clinical duties, of all true healers. This law, with its corollaries, admits no addition to its own proper means, which it declares to be sufficient for the cure of all which is curable of human suffering. It rejects all of the indirect means which have been used by some with the thought that these somehow aid the action of the specific remedy, and contribute beneficially to the expected cure. The experience of our friend and that of all most skillful healers has abundantly shown that all such means are always and only a detriment to the success which the law promises to a strict compliance with its demands. The law otherwise taught can only memorialize Adolph Lippe's disgust and wrath when he saw the law he loved so trangressed and abused.

The teaching which is to memorialize our deceased friend and his work must plainly set forth the dynamic nature of both sickness and the medicines by which they are cured. It must be clearly shown that sicknesses are not material *things*, but ever and only perverted functions of one or more bodily organs, as these have been modified by the actions of some noxia on the life-force which moves and controls these functions, this noxia and this force being also dynamic in their nature, and not material entities at all. It is absolutely necessary that the nature be recognized by the prescriber before he can parallel Adolph Lippe's successes, or comprehend the true nature of the elements of any problem of healing.

The dynamic nature of the healing factor is no less a truth of the greatest importance, which is to be well understood, and authoritatively present in the mind of the prescriber before he can intelligently take the first step in the duties of specific medicine. The discovery of this nature was wholly Hahnemann's. He not only discovered this dynamic nature of the healing factor, but also the no less important fact that this dynamic could be separated from its drug operation, expanded, and its efficient action on the living organism greatly intensified by a manipulation which he was the first to teach. It is not forgotten that this great and amazing truth has been denounced and ridiculed as one of Hahnemann's fallacies by those, and they have been many, who have never emerged from the obscurity of materialism. They have not come to, nor accepted, the light which the dynamization of the healing factor has thrown on the clinical experiences of specific medicine. Nor is it forgotten that truth is no less truth after ridicule than it was before. It is well remembered that neither ridicule, laughter, negation, nor hate have power over truth to inflict on it the least injury. Truth always has the mastery in the end, and controls affairs after laughter has died away, and with it those also who have ridiculed and hated. The Almighty has stamped eternity on truth, and, therefore, neither ignorance nor arrogance nor presumption can have power over it. It stands before them and their work in all its dignity, and calm, unmoved, and unscathed by whatever of malice, falsehood or hate of its enemies. It so stands because it is the child of God.

An organization which shall clearly teach the philosophical elements of specific medicine as we have endeavored briefly to outline them, will most successfully bring Adolph Lippe to the memory of man. These elements were the fundamental principles which inspired, directed, and controlled his life work. He took these into his heart, at the beginning from the *great master*, whom he revered and loved, and in all his work of teaching and healing he was loyal to them to the end. It was this singular loyalty which assured to him the great success which characterized his work, and made him quite illustrious in the first triumph of his life. If he can still have cognizance of the affairs which are going on in the world which he has left, what could give him greater joy than to see those who are to be the successors in his life work receiving instruction in the principles of philosphy and practice which he had accepted, believed, and loved; to know these principles were to be presented in the confidence, love, and work of the rising generation of those who will be taught to emulate his example, and strive by the means he himself employed to attain to his successors?

In our last words, we would say to the ladies of the Association, and to those who are connected with the organization from which this memorial teaching is to come, no greater blessing can come to any than will flow in on you, when this organization you have created shall be found successfully carrying on the work your love and generosity and love of truth have planned. The love of truth, wherever it has attempted to give active, practical existence for the good of mankind, has always met difficulties and opposition before it ripened into full life and action. Its progress in its way to perfect development has had to meet and overcome opposition and perhaps falsehood at each step in its progress. Let it be remembered that truth, loyally sustained by its lovers, is sure to triumph over these in the end, because of its origin in the Omniscient will, and its foundation in the throne of the Infinite. In this presence you may safely persevere, knowing that in the end, whatever and whoever opposes, it will be triumphant, and in it and in the divine approval you will find exceeding great reward in your good work.

